# Evaluation of Tuberculosis Underreporting in Greece through Comparison with Anti-Tuberculosis Drug Consumption

**DOI:** 10.1371/journal.pone.0050033

**Published:** 2012-11-21

**Authors:** Theodore Lytras, Georgia Spala, Stefanos Bonovas, Takis Panagiotopoulos

**Affiliations:** 1 National School of Public Health, Athens, Greece; 2 Hellenic Centre for Disease Control and Prevention, Athens, Greece; Johns Hopkins Bloomberg School of Public Health, United States of America

## Abstract

Surveillance is an integral part of tuberculosis (TB) control. Greece has a low TB notification rate, but there are doubts about underreporting. Examining anti-TB drug consumption is a way to validate the results of surveillance and estimate TB burden in the country. We used surveillance data from 2004 to 2008 to calculate the average prescribed treatment duration with the first-line anti-TB drugs isoniazid, rifampicin, ethambutol and pyrazinamide. We then obtained the best available data on consumption of these drugs, and calculated the number of treated cases to which these quantities correspond. We thus estimated underreporting at around 80% (77–81%), and annual TB incidence at about 30 cases per 100,000 population, five times over the notification rate. Underreporting was found to be constant over the study period, while incidence followed a decreasing trend. In addition we estimated that one person receives chemoprophylaxis for latent tuberculosis infection (LTBI) for every three TB cases. These results indicate the need for a comprehensive plan to improve TB surveillance and TB contact tracing in Greece, especially in light of the economic crisis affecting the country since 2009.

## Introduction

Tuberculosis (TB) is a major global public health concern [1], and surveillance is an integral part of any TB control plan [2]–[3], including the Directly Observed Treatment Short-course (DOTS) framework [4]. Mandatory notification of cases is the basis of most TB surveillance systems, but it can be affected by underreporting in both high- and low-prevalence countries [5]–[8]. As a result, notification rates might not accurately reflect TB incidence and comparisons between countries become less straightforward.

In Greece, TB has been a notifiable disease since 1950 and the notification rate is currently one of the lowest in Europe [9]. However, previous studies have suggested significant underreporting exists [10]–[11]. These previous studies were either undertaken at a regional level [10], were too old [11], or assessed anti-TB drug consumption in isolation [11]. We undertook this study in order to estimate and quantify TB underreporting, at the national level, by comparing surveillance data with anti-TB drug consumption data. This is possible because anti-TB drugs are highly specific for the treatment of TB (with the major exception of INH, which is also used for chemoprophylaxis of latent tuberculosis infection), are recommended for the great majority of TB cases, and are mostly used in standardized treatment regimens [12]. Our aim was also to demonstrate a simple and convenient method to estimate completeness of TB notification.

In the Greek surveillance system, physicians and/or laboratories are obliged to report TB cases at the time of diagnosis. A notification form is sent to either the Public Health Directorate of the local prefecture (PHD), or directly to the Hellenic Centre for Disease Control and Prevention (HCDCP). The 51 PHDs and HCDCP share data; the PHDs are responsible for local public health action, such as TB contact tracing, while HCDCP monitors TB epidemiology and trends at the national level. Cases (new or recurrent) are notified at the time of diagnosis, and are classified as “possible”, “probable” and “confirmed” according to the ECDC/European Commission case definition [13]. Νo treatment outcome monitoring is performed as part of TB surveillance; drug resistance monitoring is carried out by a small number of laboratories [14].

The Greek National Organization for Medicines (NOM) collects yearly data from all pharmaceutical companies active in the country about all drug quantities dispensed to hospitals and wholesalers nationwide. Essential drugs that are not available for a period of time from any company, are imported through the state Institute of Pharmaceutical Research and Technology (IPRT), which also keeps track of all quantities. These two sources are independent and complementary, and their combined data can be used as an approximate measure of total national drug consumption, with two reservations: drug wastage and parallel exports. Drug wastage in Greek hospitals has not been studied to date, but it is believed to be a very small amount. Parallel export of drugs has been a problem for Greece, due to the low regulated prices, and sometimes results in drug shortages. Evidence of this is only anecdotal, in news sources; drugs reported to be often targeted for export are mainly expensive and widely-used medications such as antihypertensive, antidiabetic, anti-cancer and neuropsychiatric drugs. There has never been any report of parallel exports of anti-TB drugs, and given their low price and narrow therapeutic scope such a possibility is highly unlikely.

The primary objective of the current study was to estimate underreporting and incidence of TB in Greece (corrected for underreporting) for the period 2004–2008, by comparing surveillance data with anti-TB drug consumption data. The study period is just prior to the current economic crisis affecting Greece since 2009. Secondary objectives of the study were to estimate annual trends in underreporting and TB incidence during this period, and the number of TB cases receiving isoniazid chemoprophylaxis for Latent Tuberculosis Infection (LTBI).

## Methods

We focused on the TB cases notified to HCDCP as part of routine surveillance between 2004 and 2008. We used treatment prescription data on these cases to calculate the average prescribed treatment duration with each of the four first-line anti-TB drugs: isoniazid (INH), rifampicin (RMP), ethambutol (EMB) and pyrazinamide (PZA). All notified cases were in the denominator, including those that were not prescribed any of these drugs. In cases where the exact treatment regimen was not clear from the notification form and deviated from the standard regimens used [12], we assumed that each anti-TB drug was prescribed for the entire duration of treatment. As treatment outcome data were not available, we only considered interruption of treatment in cases where the patient died or defaulted before notification was made.

Consumption data for the four anti-TB drugs were obtained from NOM and IPRT. We combined these quantities and calculated for each drug the total number of Defined Daily Doses (DDDs) dispensed between 2004 and 2008 (Table 1). DDD is a unit of measurement defined by the WHO collaborating centre for Drug Statistics Methdology, which corresponds to the average daily dosage for the drug's main indication in adults with normal organ function [15]. It is used for drug consumption research and allows meaningful comparisons to be made. It was determined that the DDD for anti-TB drugs closely matches the dosage recommended by HCDCP and NOM [16]–[17] (table 1), which is also, to our experience, the average daily dosage usually prescribed by clinicians in our country. Thus we considered the total number of DDDs for each drug as an estimate of the total person-time of treatment with the respective drug.

**Table 1:** Defined Daily Dose (DDD) [15] and recommended dose for anti-TB drugs [16]–[17].

Anti-TB drug	Defined Daily Dose (DDD)	Recommended daily dose in Greece
Isoniazid (INH)	300 mg	5 mg/kg, up to 300 mg
		Children: 10–20 mg/kg, up to 300 mg
Rifampicin (RMP)	600 mg	10 mg/kg, up to 600 mg
		Children: 10–20 mg/kg, up to 600 mg
Ethambutol (EMB)	1200 mg	15 mg/kg
		Children: 15–20 mg/kg
Pyrazinamide (PZA)	1500 mg	25–35 mg/kg/day, up to 2 g
		Children: 20–30 mg/kg

We assumed that there is no difference in treatment prescribed between TB cases notified and those not notified. Therefore we obtained independent estimates (one for each anti-TB drug) of the total number of TB cases by dividing the total DDDs dispensed by the average treatment duration (in days). Similarly we estimated underreporting by: 1– (cases notified) * (average treatment duration)/(total DDDs dispensed).

We estimated TB incidence and underreporting both for the entire study period (2004–2008) and for each single year. For the incidence calculations, we used the mid-year populations projected by the Greek Statistical Authority (ELSTAT) based on the 2001 census. We used all four primary anti-TB drugs to obtain seperate estimates for the entire study period. Annual estimates were obtained with EMB and PZA only; such estimates based on INH and RMP would not be statistically independent, because treatment with these drugs is much longer and would span two consecutive years in a lot of cases. We used regression models to examine trends, with TB incidence and underreporting as dependent and year of notification as independent variables [18].

We estimated a range for the number of persons treated for LTBI calculating the excess quantity of INH dispensed in comparison to RMP, and assuming each person was treated for a minimum of six to a maximum of nine months, in line with current recommendations [12] [16].

All data processing and calculations were done using the GNU R software environment, version 2.12.

## Results

**Figure 1:**
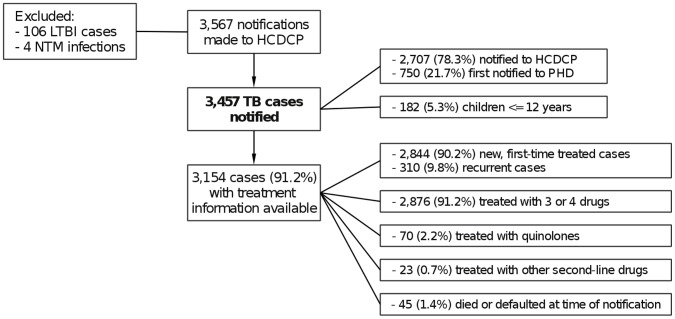
Characteristics of notified TB cases. Abbreviations: LTBI – Latent Tuberculosis Infection, NTM – Non-tuberculous Mycobacteria, HCDCP – Hellenic Centre for Disease Control and Prevention, TB – Tuberculosis, PHD – Prefectural Public Health Department.

During the 5-year study period (2004–2008) there was a total of 3,567 TB notifications made to HCDCP (Figure 1). We went through all notification forms one by one and tried to exclude any cases that clearly did not meet the case definition for TB. We identified four cases of non-tuberculous mycobacteria (NTM) infections, as well as 106 cases of LTBI. These were excluded from further analysis. Therefore, during the study period there was a total 3,457 TB cases notified to HCDCP, corresponding to an average annual notification rate of 6.2 cases per 100,000 population (table 2). 750 (21.7%) of these cases had been notified to the prefectural PHD first, while the remaining 2,707 cases (78.3%) were notified directly to HCDCP. 182 cases (5.3%) were children up to 12 years old. For 3,154 cases (91.2%) the treatment regimen was specified in the case notification form. Of those, 2844 (90.2%) were treated for the first time, while 310 (9.8%) were recurrent cases. Although TB cases must be notified at the time of diagnosis, in our data notification occured after treatment initiation by a mean of 14.7 days (SD 19.8 days); as a result, 45 cases (1.3%) had died or defaulted on their treatment at the time of notification. The large majority (91.2%–2,876 cases) had been prescribed treatment with three or four drugs, 2.2% (70 cases) with quinolones and 0.7% (23 cases) with other second-line drugs.

**Table 2:** Tuberculosis notifications in Greece, 2004–2008.

	Number of TB cases notified	Notification rate per 100,000 population
2004	753	6.8
2005	769	6.9
2006	636	5.7
2007	639	5.7
2008	660	5.9
Total	3,457	6.2

Rate calculated using the mid-year populations projected by the Greek Statistical Authority (ELSTAT, www.statistics.gr), based on the 2001 census [27].

The notified TB cases for whom treatment regimen was specified (n = 3,154) had been prescribed treatment for a mean 7.75 months (236 days, SD 67 days) with INH, 7.80 months (237 days, SD 68 days) with RMP, 2.34 months (71 days, SD 56 days) with EMB and 1.59 months (48 days, SD 39 days) with PZA (table 3). The mean prescribed treatment duration with EMB and PZA by year is shown on tables 4 and 5.

**Table 3:** Estimated number of total tuberculosis (TB) cases, incidence rate and underreporting, per major anti-TB drug, Greece, 2004–2008.

Anti-TBdrug	Mean prescribedtreatment duration, inmonths (95% CI)	DDDsdispensed	Estimated total TBcases (95% CI)	Estimated annual incidencerate, per 100,000population (95% CI)	Estimatedunderreporting(95% CI)
INH	7.75 (7.70–7.80)	5,383,293	22,849 (22,702–22,998)	41 (40.7–41.2)	84.9% (84.8%–85%)
RMP	7.80 (7.75–7.85)	4,233,888	17,855 (17,742–17,971)	32 (31.8–32.2)	80.6% (80.5%–80.8%)
EMB	2.34 (2.28–2.41)	1,243,887	17,479 (17,011–17,972)	31.4 (30.5–32.2)	80.2% (79.7%–80.8%)
PZA	1.59 (1.55–1.64)	748,416	15,465 (15,045–15,909)	27.7 (27.0–28.5)	77.6% (77.0%–78.3%)

**Table 4:** Annual estimated number of total tuberculosis (TB) cases, incidence rate and underreporting, on the basis of ethambutol (EMB) consumption, Greece, 2004–2008.

Year	Mean prescribed treatment duration, in months (95% CI)	Estimated total TB cases(95% CI)	Estimated annual incidence rate, per 100,000 population (95% CI)	Estimated underreporting(95% CI)
2004	2.27 (2.14–2.40)	3,710 (3,509–3,936)	33.5 (31.7–35.6)	79.7% (78.5%–80.9%)
2005	2.37 (2.24–2.50)	3,864 (3,658–4,095)	34.8 (32.9–36.9)	80.1% (79.0%–81.2%)
2006	2.50 (2.32–2.68)	3,249 (3,035–3,495)	29.1 (27.2–31.3)	80.4% (79.0%–81.8%)
2007	2.29 (2.16–2.41)	3,316 (3,141–3,513)	29.6 (28.1–31.4)	80.7% (79.7%–81.8%)
2008	2.29 (2.14–2.44)	3,344 (3,138–3,578)	29.8 (27.9–31.8)	80.3% (79.0%–81.6%)
Total	2.34 (2.28–2.41)	17,479 (17,011–17,972)	31.4 (30.5–32.2)	80.2% (79.7%–80.8%)

Weighted exponential regression coefficients (incidence rate): e^β^ = 0.96, 95% CI 0.91–1.02, p = 0.11. Linear regression coefficients (underreporting): β = +0.2%, 95% CI −0.1% to +0.5%, p = 0.15.

**Table 5:** Annual estimated number of total tuberculosis (TB) cases, incidence rate and underreporting, on the basis of pyrazinamide (PZA) consumption, Greece, 2004–2008.

Year	Mean prescribed treatment duration, in months (95% CI)	Estimated total TB cases(95% CI)	Estimated annual incidence rate, per 100,000 population (95% CI)	Estimated underreporting(95% CI)
2004	1.29 (1.19–1.39)	4,365 (4,053–4,730)	39.5 (36.6–42.8)	82.7% (81.4%–84.1%)
2005	1.49 (1.39–1.59)	3,595 (3,374–3,848)	32.4 (30.4–34.7)	78.6% (77.2% - 80.0%)
2006	1.76 (1.66–1.87)	2,806 (2,654–2,975)	25.2 (23.8–26.7)	77.3% (76.0%–78.6%)
2007	1.67 (1.57–1.77)	2,689 (2,538–2,859)	24.0 (22.7–25.5)	76.2% (74.8%–77.6%)
2008	1.78 (1.69–1.88)	2,359 (2,241–2,491)	21.0 (19.9–22.2)	72.0% (70.5%–73.5%)
Total	1.59 (1.55–1.64)	15,465 (15,045–15,909)	27.7 (27.0–28.5)	77.6% (77.0%–78.3%)

Weighted exponential regression coefficients (incidence rate): e^β^ = 0.86, 95% CI 0.80–0.92, p = 0.005. Linear regression coefficients (underreporting): β = −2.4%, 95% CI −3.5% to −1.3%, p = 0.006.

The total quantity of the four anti-TB drugs dispensed from 2004 to 2008 appears on table 6. On linear regression there was a statistically significant downward trend in the consumption of PZA, while the trend was not significant for the other drugs.

**Table 6:** Anti-TB drug consumption in Greece, 2004–2008, in Daily Defined Doses (DDDs).

Year	Isoniazid(INH)	Rifampicin(RMP)	Ethambutol(EMB)	Pyrazinamide(PZA)
2004	841,397	893,352	255,800	171,209
2005	938,264	892,341	278,220	162,730
2006	1,163,189	733,334	246,917	150,470
2007	1,303,390	860,013	230,367	136,285
2008	1,137,053	854,848	232,583	127,722
Total	5,383,293	4,233,888	1,243,887	748,416

Linear regression coefficients: INH: β = 95,644, p = 0.09, R^2^ = 0.56; RMP: β = −10,934, p = 0.67, R^2^ = −0.24; EMB: β = −9,429, p = 0.13, R^2^ = 0.47; PZA: β = −11,342, p<0.001, R^2^ = 0.99.

Based on these data, table 3 shows our results for the entire study period 2004–2008. With the exception of INH, the estimates for each outcome are fairly close to each other; underreporting is estimated at around 80% (77–81%), and annual TB incidence at around 30 cases per 100 000 population, five times higher than the notification rate.

We used EMB and PZA to obtain annual estimates for TB incidence and underreporting (tables 4 and 5). On weighted exponential regression, TB incidence showed an average decrease of 14% every year (e^β^ = 0.86, 95% CI 0.80–0.92, p = 0.005) when estimated on the basis of PZA, and a smaller decreasing trend of 4% per year (e^β^ = 0.96, 95% CI 0.91–1.02, p = 0.11) when estimated on the basis of EMB. Underreporting was constant when estimated on the basis of EMB (β = +0.2%, 95% CI −0.1% to +0.5%), and appeared to decrease minimally when estimated on the basis of PZA (β = −2.4%, 95% CI −3.5% to −1.3%).

As the mean treatment duration with INH and RMP was practically the same (table 3), we considered that the excess quantity of INH dispensed (5,383,293–4,233,888 = 1,149,405 DDDs) corresponds to usage for chemoprophylaxis of LTBI cases. For treatment of LTBI, INH is administered for 6 to 9 months [12] [16]; for example, the 106 cases of LTBI that were notified to HCDCP as TB cases and were eliminated from our dataset, received a mean of 7.5 months of INH treatment. Therefore this quantity corresponds to a minimum of 4,257 and a maximum of 6,386 LTBI cases treated between 2004 and 2008. During the same period the estimated total TB cases ranged from 15,045 to 17,972 cases (table 3). Therefore the average ratio of treated LTBI cases per TB case was estimated between 0.24 and 0.42, i.e. one person receives INH chemoprophylaxis for approximately every three TB cases.

## Discussion

Our study used anti-TB drug consumption data to validate surveillance data and estimate underreporting and TB incidence. Underreporting of TB cases in Greece for the period 2004–2008 was estimated at around 80% (77–81%), corresponding to an incidence of about 30 cases per 100,000 population per year, five times higher than the notification rate. This should be interpreted in light of the underlying assumptions, as an approximate estimate reflecting the level of magnitude of the problem. It is difficult to attribute this degree of underreporting solely to regional PHDs not forwarding their data to HCDCP; rather it is probably due to clinicians having a low awareness of the importance of TB notification.

Previous studies in Greece demonstrated an underreporting of 66% nationwide in 1984–1988 [11], and 73% in one region in 2000–2003 [10]; our study suggest a somewhat higher level. Our study also suggests a decreasing trend in TB incidence (4% per year on the basis of EMB), which is consistent with the trend observed both in surveillance data (Table 2) and in recent data published by the Greek National Reference Laboratory for Mycobacteria [14]. Underreporting remained relatively stable during the study period. This indicates that although surveillance data considerably underestimate the level of TB incidence, they satisfactorily depict its trend overtime during this period.

An interesting finding of our study is the low number of treated LTBI cases per TB case, several times lower than in other surveys (0.24–0.42 vs. 1.2–1.9) [19]–[22]. This discrepancy may be explained by inadequacies in contact tracing capacity and policies of public health services in Greece. It may also be explained by the higher level of underreporting in Greece, since contact tracing is unlikely to follow if a case is not notified. The observed increase in INH consumption during the study period (table 6) may suggest some improvement in TB contact tracing in recent years, though still lagging behind the rate found in other surveys [19]–[22].

Previous studies with similar methodology have used only PZA consumption [23]–[24]; we used all four first-line drugs (INH, RMP, EMB and PZA) as a basis for our estimates, and obtained fairly consistent results. The exception is INH, which apart from TB treatment also has the major indication for treatment of LTBI. Thus INH-based estimates for TB incidence and underreporting are expected to be biased upwards, and essentially represent an upper limit. With the other drugs, different sources of error can affect our estimates. RMP is very specific for treatment of TB and is included in almost all treatment regimens. The duration of RMP treatment can vary, however, thus there is more margin for error in the mean treatment duration calculated from the notified TB cases (Table 3). RMP is administered for a longer period than EMB and PZA, therefore there is bigger potential for treatment default. In addition there are a few minor indications for RMP other than TB treatment, which could also lead to a slight overestimation of our results; these include prophylaxis for meningococcal meningitis, and second-line treatment of Brucellosis and Legionnaire's disease. EMB and PZA are exclusively used for treating TB, and their treatment duration is shorter, thus there is less potential treatment default. However, especially with PZA, there is bigger variability in individual drug dosage. Variation in dosing practices can be a significant source of error in our estimates (in either direction) [25]. As a result, it is important to compare estimates with more than one drug, to increase reliability.

Another discrepancy in our estimates is the trend observed with PZA: a big decrease of 14% per year in TB incidence, and a decrease in TB underreporting by 10% from 2004 to 2008. These results are influenced by a lower average treatment duration with PZA for 2004 and 2005, which contrasts with a higher PZA consumption during these two years. In essence, TB cases notified in 2004 and 2005 were less likely to be treated with PZA than cases notified in other years. The reason for this finding is not clear, but it may be down to heterogenous treatment practices among Greek hospitals, along with constantly changing notification rates from each hospital. In any event, the trends estimated on the basis of PZA should be approached with caution.

Our study has various limitations. First, the study design does not allow a direct link to be made between drug use and individual TB patients; therefore the results should be interpreted with caution. Another significant limitation is the unavailability of treatment outcome monitoring data; as a result, treatment default and loss to follow-up beyond initial notification were not assessed, and this would lead to an underestimate of TB incidence and underreporting. Similarly, we could not assess whether clinicians in some cases extend treatment duration beyond the time specified in the notification form. This would lead to some degree of overestimation.

Due to lack of precise case-level treatment data from TB surveillance, we used the number of DDDs as a proxy measure for person-time of treatment. Potential deviations of the DDD from the actual mean prescribed dosage can be a source of error. Because only a small proportion of reported cases were children (5.3% were 12 years old or less), we did not account for the lower anti-TB drug dosage in this age group; this can lead to an underestimate of TB underreporting and incidence. Another limitation concerns the data from NOM and IPRT; these are not actual patient consumption data, but rather market dispensation data. As a result, although unlikely, we cannot exclude the possibility of parallel exports, significant drug wastage or any other source of unconsumed anti-TB drugs, which would lead to overestimation of our results.

In addition, the current study is not suitable to assess any systematic differences in treatment between reported and non-reported TB cases, any heterogeneity of treatment practices in various groups of cases, or any specific patterns of underreporting. Nor is the study designed to address underdiagnosis of TB, e.g. in special groups and deprived parts of the population.

Despite these limitations, our results indicate that this is an appropriate and convenient method to approach the question of TB underreporting, and they effectively underescore the weaknessess in the Greek TB surveillance system. Further research is needed to quantify the burden of TB in Greece more accurately; ideally a record linkage and/or capture-recapture study should be performed in order to validate the estimates of the current study.

This nationwide evaluation of TB surveillance in Greece addresses the period before the current economic crisis affecting the country. The crisis is expected to present additional challenges for the surveillance system, and highlights the need to address its shortcomings. To that end, the experience of other developed countries can be useful [26]. The importance of notifying TB cases needs to be proactively advertised to clinicians. Emphasis must be placed on treatment outcome monitoring and on data from TB laboratories, in order to identify unreported cases and monitor drug resistance. In addition, TB surveillance may be coupled with case management and include information such as socioeconomic determinants. These and other ideas need to be carefully considered, as improving surveillance is paramount for the overall TB control effort in Greece.
